# Effects of Melanocortin 3 and 4 Receptor Deficiency on Energy Homeostasis in Rats

**DOI:** 10.1038/srep34938

**Published:** 2016-10-07

**Authors:** Panpan You, Handan Hu, Yuting Chen, Yongliang Zhao, Yiqing Yang, Tongtong Wang, Roumei Xing, Yanjiao Shao, Wen Zhang, Dali Li, Huaqing Chen, Mingyao Liu

**Affiliations:** 1 Shanghai Key Laboratory of Regulatory Biology, Institute of Biomedical Sciences, Shanghai 200241, China; 2 School of Life Sciences, East China Normal University, Shanghai 200241, China; 3 Institute of Biosciences and Technology, Department of Molecular and Cellular Medicine, Texas A&M University Health Science Center, Houston, Texas 77030, USA

## Abstract

Melanocortin-3 and 4 receptors (MC3R and MC4R) can regulate energy homeostasis, but their respective roles especially the functions of MC3R need more exploration. Here *Mc3r* and *Mc4r* single and double knockout (DKO) rats were generated using CRISPR-Cas9 system. Metabolic phenotypes were examined and data were compared systematically. *Mc3r* KO rats displayed hypophagia and decreased body weight, while *Mc4r* KO and DKO exhibited hyperphagia and increased body weight. All three mutants showed increased white adipose tissue mass and adipocyte size. Interestingly, although *Mc3r* KO did not show a significant elevation in lipids as seen in *Mc4r* KO, DKO displayed even higher lipid levels than *Mc4r* KO. DKO also showed more severe glucose intolerance and hyperglycaemia than *Mc4r* KO. These data demonstrated MC3R deficiency caused a reduction of food intake and body weight, whereas at the same time exhibited additive effects on top of MC4R deficiency on lipid and glucose metabolism. This is the first phenotypic analysis and systematic comparison of *Mc3r* KO, *Mc4r* KO and DKO rats on a homogenous genetic background. These mutant rats will be important in defining the complicated signalling pathways of MC3R and MC4R. Both *Mc4r* KO and DKO are good models for obesity and diabetes research.

The melanocortin system plays an important physiological role in energy homeostasis^1^. Melanocortin-3 and 4 receptors (MC3R and MC4R) are two neural melanocortin receptors that belong to the rhodopsin-like G protein-coupled receptor family. MC4R is expressed throughout the central nervous system (CNS)^2^, and mutations in the MC4R gene are the most common cause of monogenic obesity in human^3^,^4^,^5^,^6^. Many researchers reported that MC4R participates in regulating feeding behavior and energy expenditure^7^,^8^. In addition, MC4R is expressed in peripheral such as adipose tissue^9^. Recently it was recognized as the second most highly expressed G protein-coupled receptor in enteroendocrine L cells, which suggests that peripheral MC4R is important in specific physiological functions^10^. MC3R is also expressed in CNS especially in the arcuate nucleus, as well as some peripheral tissues such as heart and peritoneal macrophages^11^,^12^,^13^,^14^. Studies in human and rodents have confirmed MC3R expression in the kidney, where it participates in modulation of natriuresis^13^,^15^. Importantly, MC3R has been found to play a subtle role in regulating energy homeostasis^12^,^16^. There are also some reports about the influence of variant *Mc3r* alleles on human obesity^17^,^18^,^19^, especially the recent paper about replacing mouse locus with human obesity related MC3R double-mutant suggesting an important role of MC3R in human metabolism^20^.

*Mc4r* and *Mc3r* knockout (KO) mouse models have provided some evidence for their respective role in energy homeostasis. *Mc4r* KO mice exhibited obesity, hyperphagia, hyperglycaemia and hyperinsulinemia^8^,^21^, while *Mc3r* KO mice displayed almost the same body weight as WT control up to 26 weeks of age, but they did exhibit a mild obesity phenotype characterized by increased fat mass, reduced lean mass and reduced body length^16^. Although not much data is available about *Mc4r*/*Mc3r* double KO (DKO) mice, they were reported to be significantly more obese than *Mc4r* KO mice^16^. As genetic background can significantly influence the phenotype of obesity^22^,^23^, a true phenotypic comparison among *Mc3r* KO, *Mc4r* KO and DKO in the same congenic background would give more valuable information.

The laboratory rat is a valuable mammalian model organism for many human diseases and has advantages over the mouse such as in metabolic and pharmacological studies. The first functional knockout rat (Wistar/Crl background) for *Mc4r* (Mc4r^K314X^) was reported in 2011 as the result of an N-ethyl-N-nitrosourea-induced point mutation^24^, and exhibited increased body weight, food intake and white adipose mass, and altered substrate preference^25^. In 2013 we published the generation of *Mc4r* KO, and *Mc4r/Mc3r* DKO rat by CRISPR-Cas9 and observed a similar phenotype of the *Mc4r* KO rat in body weight, food intake, insulin and leptin level as compared to the N-ethyl-N-nitrosourea-induced *Mc4r* KO rat, although these observations were only based on founders with biallelic mutants^26^. Meanwhile, no report on phenotypic analysis of the *Mc3r* KO rat or *Mc3r/Mc4r* DKO rat was found. In this study, an investigation on *Mc3r KO, Mc4r* KO, and DKO rats was carried out and data were compared systematically regarding their metabolic phenotypes in a single genetic background, in order to provide an overall understanding of these two receptors in energy metabolism, and to evaluate the possibility of using these KO rats as suitable animal models for obesity or type 2 diabetes. Results showed that MC4R deficiency led to a dramatic alteration in energy homeostasis including obesity and hyperglycaemia, whereas MC3R deficiency showed hypophagia and reduced body weight. However, MC3R deficiency appeared to exert an additive effect on MC4R deficiency in several aspects including lipid profile and glucose metabolism which implies that different pathways mediated MC3R’s effect on different functions. Data from our extensive phenotypic comparison suggested that *Mc4r* KO and DKO might be good rat models for obesity or type 2 diabetes (T2D).

## Results

### Effect of MC3R and MC4R deficiency on growth curve, size and Lee’s index

**Figure 1:**
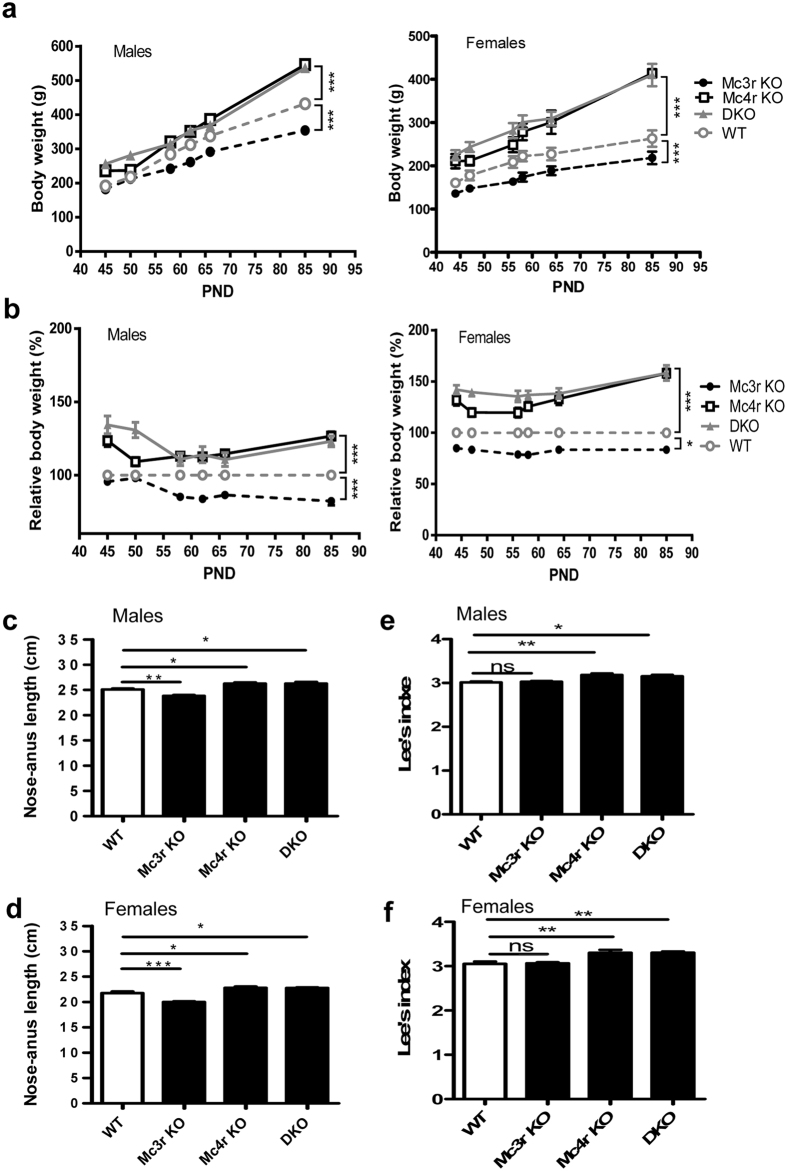
Effect of MC4R and MC3R deficiency on body weight, body length and Lee’s index. (**a**) Body weight and (**b**) Relative body weight were measured from postnatal day (PND) 45 to 85 for *Mc3r* KO, *Mc4r* KO, DKO and WT littermates (n = 8 except 6 for DKO, repeated measures analysis followed by Bonferroni post-tests). Nose-anus length (**c,d**) and Lee’s index (**e,f**) of *Mc3r* KO, *Mc4r* KO, and DKO rats vs. WT littermates (n = 8 except 4 for DKO) at 14 weeks of age (one-way ANOVA test). Data are shown as mean ± SEM. *p < 0.05, **p < 0.01, ***p < 0.001 vs. WT control.

Heritable total *Mc3r* and *Mc4r* knockout (KO) rats were generated using CRISPR-Cas9 system in Sprague Dawley (SD) rats^26^. DNA sequences of *Mc3r* or *Mc4r* genomic loci in mutant rats were verified as correct. The expression of *Mc3r* and *Mc4r* in liver, brain and visceral total white adipose tissue (WAT) of mutants reduced significantly as compared with WT (Supplementary Fig. S1). This indicates that premature stop codons decreased mutant mRNA stability, as it is well established that frameshift or nonsense mutations induce mRNA decay^27^. All *Mc3r, Mc4r* single KO and double KO rats appeared normal phenotypically except for changed body size. We monitored the body weight of male and female *Mc3r* KO and *Mc4r* KO rats from 4–18 weeks old, and DKO rats from postnatal day (PND) 45 to 85. It was found that *Mc3r* KO displayed a significantly reduced body weight as compared to WT littermates, beginning at PND 58 in males and PND 28 in females (Fig. 1a,b, n = 8, male p = 0.0006, female p < 0.0001, Supplementary Fig. S2). Meanwhile, *Mc4r* KO and DKO showed approximate same degree of increased body weight, with the only exception at time points near PND 50 (Fig. 1a,b).

The body length of different groups of rats was measured at 14 weeks of age. As shown in Fig. 1c,d, *Mc4r* KO and DKO rats were significantly longer than WT littermates (n = 4–8, male and female p = 0.02 for *Mc4r* KO, male p = 0.02, female p = 0.04 for DKO). However, the average body length of *Mc3r* KO rats was significantly shorter (Fig. 1c,d, n = 8, male p = 0.002, female p = 0.0008), which was consistent with previous reports on mice. As an indicator of obesity, Lee’s index was calculated based on body weight and body length. No change was observed in *Mc3r* KO, while in *Mc4r* KO and DKO the same significant increase was revealed (Fig. 1e,f, n = 4–8, male p = 0.001, female p = 0.006 for *Mc4r* KO, male p = 0.01, female p = 0.005 for DKO). Thus, MC3R deficiency resulted in a shorter, lighter rat, while in *Mc4r* KO and DKO, increase in body weight was consistent with the increase in body length and Lee’s index.

### Food and water intake in *Mc3r*, *Mc4r* KO and DKO rats

**Figure 2:**
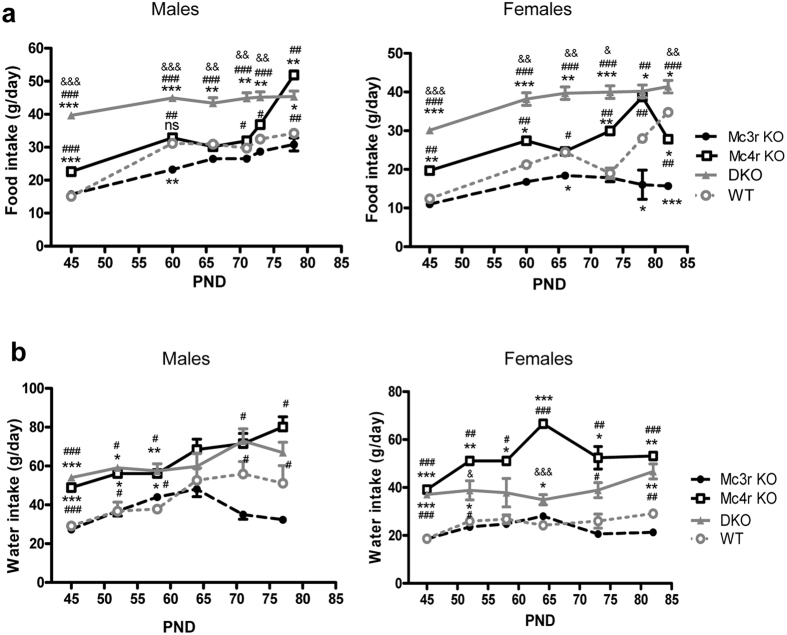
Food and water intake in different mutant rat models. (**a**) Food intake in *Mc3r* KO, *Mc4r* KO and DKO vs. WT littermates. (**b**) Water intake in *Mc3r* KO, *Mc4r* KO and DKO vs. WT littermates. Data are shown as mean ± SEM (n = 6). *Difference between mutant and WT littermates. ^#^Difference between *Mc3r* KO vs. *Mc4r* KO and DKO. ^&^Difference between *Mc4r* KO and DKO. ^#,&^ and *p < 0.05; ^##,&&^ and **p < 0.01; ^###,&&&^ and ***p < 0.001 vs. WT control (one-way ANOVA test).

Next we proceeded to monitor food and water consumption in different groups. Consistent with their body weight changes, *Mc3r* KO rats displayed a decreased food intake especially in females, as well as a reduction in water intake that did not reach significant levels as compared to WT littermates. Conversely, *Mc4r* KO and DKO had an increased food intake as well as water intake as compared to WT rats (Fig. 2). Thus the *Mc3r* KO rat was hypophagic while *Mc4r* KO and DKO rats were both hyperphagic.

### Characterization of serum lipid profile and adipose accumulation

**Figure 3:**
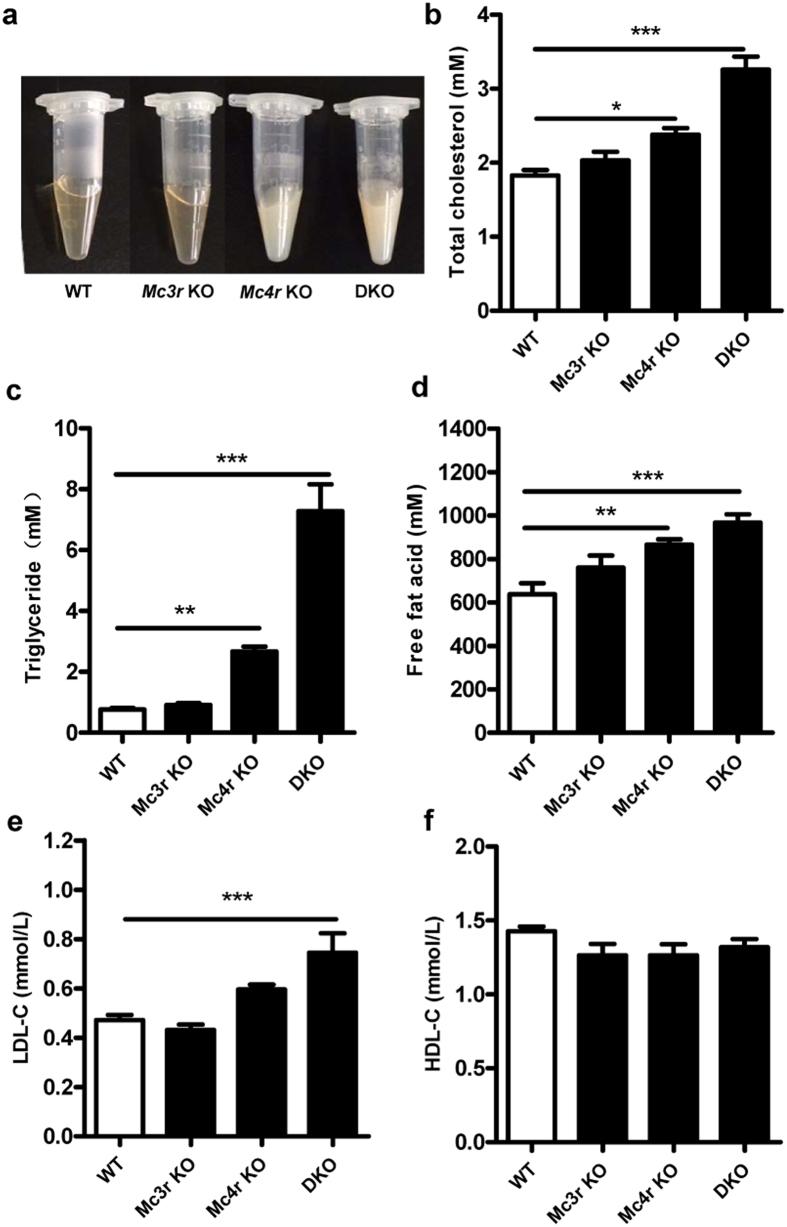
Serum lipid profile. (**a**) Typical appearance of serum from different groups of rats. (**b**) Total cholesterol (**c**) Triglyceride, (**d**) Free fatty acid, (**e**) Low-density lipoprotein cholesterol (LDL-c) and (**f**) High density lipoprotein cholesterol (HDL-c) of *Mc3r* KO, *Mc4r* KO, DKO rats and WT littermates at 8 weeks of age (n = 10–16). Data from male and female rats were merged as they showed a similar pattern and are shown as mean ± SEM. *p < 0.05, **p < 0.01, ***p < 0.001 vs. WT littermates (one-way ANOVA test).

**Figure 4:**
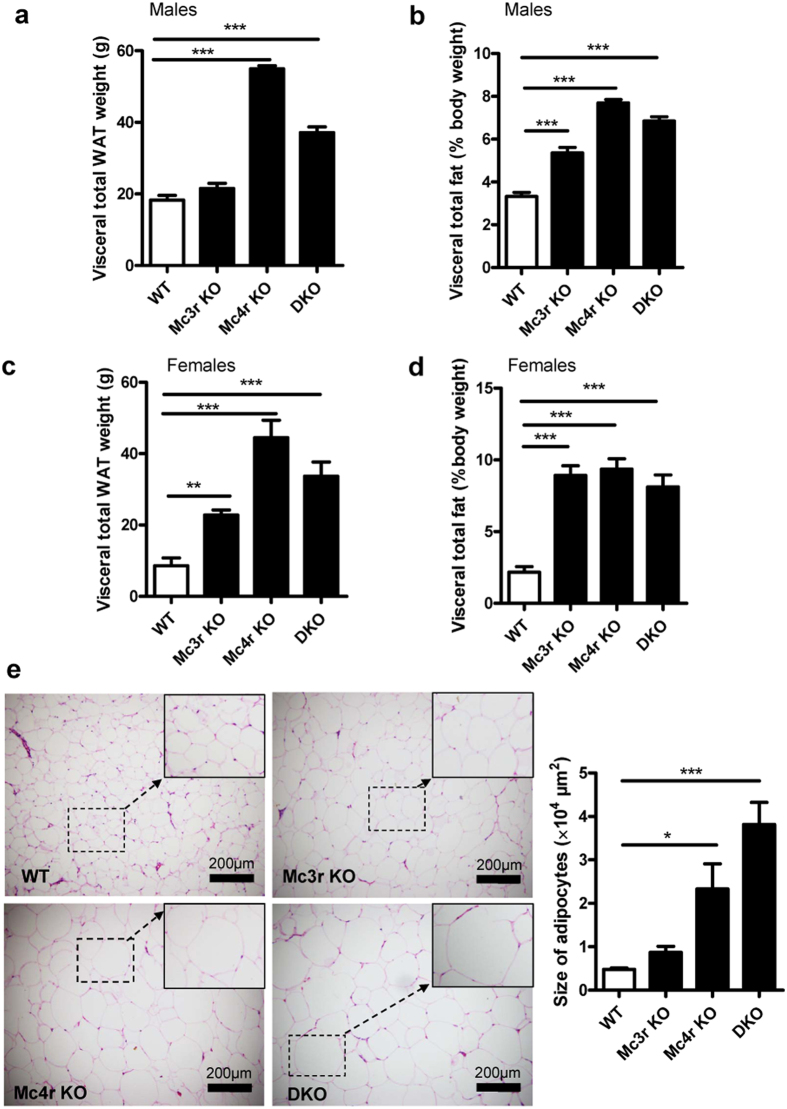
Visceral total WAT and adipocytes. Visceral total white adipose tissue (WAT) weight (**a,c**) and percentage of body weight (**b,d**) of *Mc3r* KO, *Mc4r* KO, DKO and WT controls of 14-week old male and female rats (n = 8 except 4–6 for DKO). Data are shown as mean ± SEM. *p < 0.05, **p < 0.01, ***p < 0.001 vs. WT control (one-way ANOVA test). (**e**) Representative images of adipocytes of *Mc3r* KO, *Mc4r* KO, DKO rats and WT littermates, together with quantitation (magnification ×100 and ×400), from at least 3 animals of each genotype.

Serum obtained from *Mc4r* KO and DKO rats appeared thick and milky white, obviously abnormal as compared to those of WT and *Mc3r* KO which were clear and yellowish (Fig. 3a). As expected, the lipid profile of *Mc4r* KO and DKO rats showed significantly increased total cholesterol (p = 0.01 for *Mc4r* KO, p < 0.0001 for DKO), triglyceride (p = 0.008 for *Mc4r* KO, p < 0.0001 for DKO) and free fatty acid (p = 0.007 for *Mc4r* KO, p = 0.0005 for DKO) (Fig. 3b–d, n = 10–16). Not much change was found in low-density lipoprotein cholesterol (LDL-c) and high-density lipoprotein cholesterol (HDL-c), except an increase in LDL-c was manifested in DKO rats (Fig. 3e,f). Interestingly, although *Mc3r* KO did not induce any significant alteration in lipid profile, it did show an additive effect to *Mc4r* KO on elevation of serum lipids (Fig. 3b–d). In addition, all three mutant rats displayed significantly increased WAT weight as well as percentage of body weight (Fig. 4a,b, n = 4–8, p ≤ 0.0001; Fig. 4c,d, n = 6–8, p < 0.0001). In males, the mean visceral WAT rose from 3.3% of body weight in WT to 5.4% in *Mc3r* KO, 7.7% in *Mc4r* KO, and 6.8% in DKO respectively (Fig. 4b, n = 4–8, p ≤ 0.0001). In females, it rose from 2.2% of body weight in WT to 8.9% in *Mc3r* KO, 9.4% in *Mc4r* KO, and 8.1% in DKO (Fig. 4d, n = 6–8, p < 0.0001). These differences in body fat were also reflected in adipocytes. All three mutants had an enlarged average size for adipocytes, with DKO displaying the greatest enlargement (Fig. 4e, n = 6, p = 0.05 for *Mc3r* KO, p = 0.02 for *Mc4r* KO, p = 0.0002 for DKO). Altogether, the above data showed that *Mc4r* KO and DKO had a higher serum lipid content, while *Mc3r* KO only showed an additive effect on top of *Mc4r* KO. Furthermore, all mutant rats displayed increased visceral WAT as well as enlarged adipocyte size.

### Effect of MC3R and MC4R deficiency on liver and kidney

**Figure 5:**
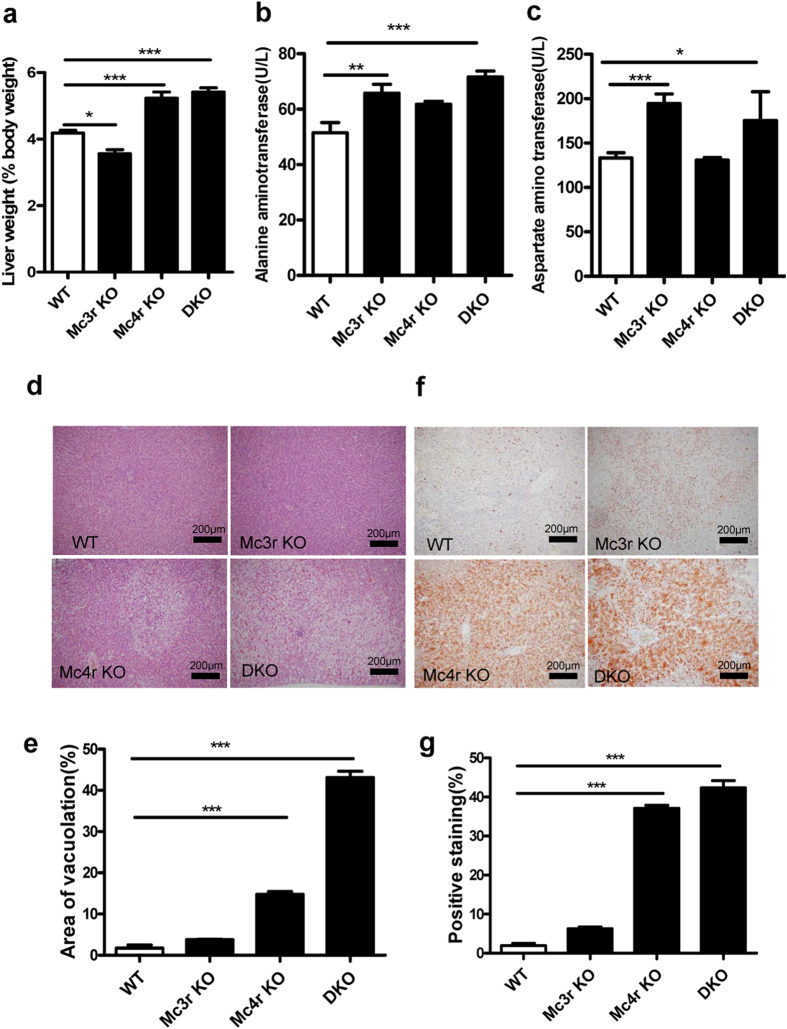
Alteration of liver in MC3R and MC4R deficient rats. (**a**) Relative liver weight (as percentage of body weight) of *Mc3r* KO, *Mc4r* KO, DKO and WT littermates at 14 weeks (n = 8). (**b**) Alanine aminotransferase and (**c**) Aspartate aminotransferase of *Mc3r* KO, *Mc4r* KO, DKO and WT littermates (n = 8). Data are shown as mean ± SEM. *p < 0.05, **p < 0.01, ***p < 0.001 vs. WT littermates (one-way ANOVA test). (**d,e**) Representative image of HE staining and (**f,g**) Oil Red-O staining of livers from at least 3 animals per group (magnification ×100). Relative areas of vacuolation or Oil Red O staining were quantified using Image-Pro^®^ Plus version 6.0 software.

Next we examined whether there was any change in the liver of mutant rats. Results showed that *Mc3r* KO rats had a relatively smaller liver, whereas *Mc4r* KO and DKO rats had significantly larger livers as compared to WT littermates (Fig. 5a, n = 8, p = 0.0005 for *Mc4r* KO, p < 0.0001 for DKO). *Mc3r* KO and DKO rats showed elevated alanine aminotransferase and aspartate aminotransferase (Fig. 5b,c). However, when we checked up hematoxylin-eosin (HE) staining of liver sections, it was obvious that the *Mc3r* KO liver was indistinguishable from WT liver, while livers of *Mc4r* KO and DKO showed accumulation of lipid in the intracellular vesicles, with the phenotype more severe in DKO livers (Fig. 5d,e). To confirm the result, we subjected liver slides to oil Red-O staining. There were significantly more red stained areas in *Mc4r* KO and DKO liver sections indicating the presence of higher level of lipids, whereas no significant change was observed in the liver of *Mc3r* KO (Fig. 5f,g, n = 6, p < 0.0001).

Effects of MC3R and MC4R deficiency on the kidney were also examined. A smaller relative kidney size was found in *Mc3r* KO and DKO rats. Elevation of uric acid was only observed in DKO. Creatinine was reduced in *Mc4r* KO. Importantly, HE staining of kidneys from KO rats did not show any significant alteration (see Supplementary Fig. S3). Therefore, MC4R deficiency led to liver steatosis but did not cause significant change in kidney histology. MC3R deficiency alone was not enough to produce a significant alteration in the liver, but it did aggravate the pathological effect of MC4R deficiency.

### Characterization of glucose metabolism

**Figure 6:**
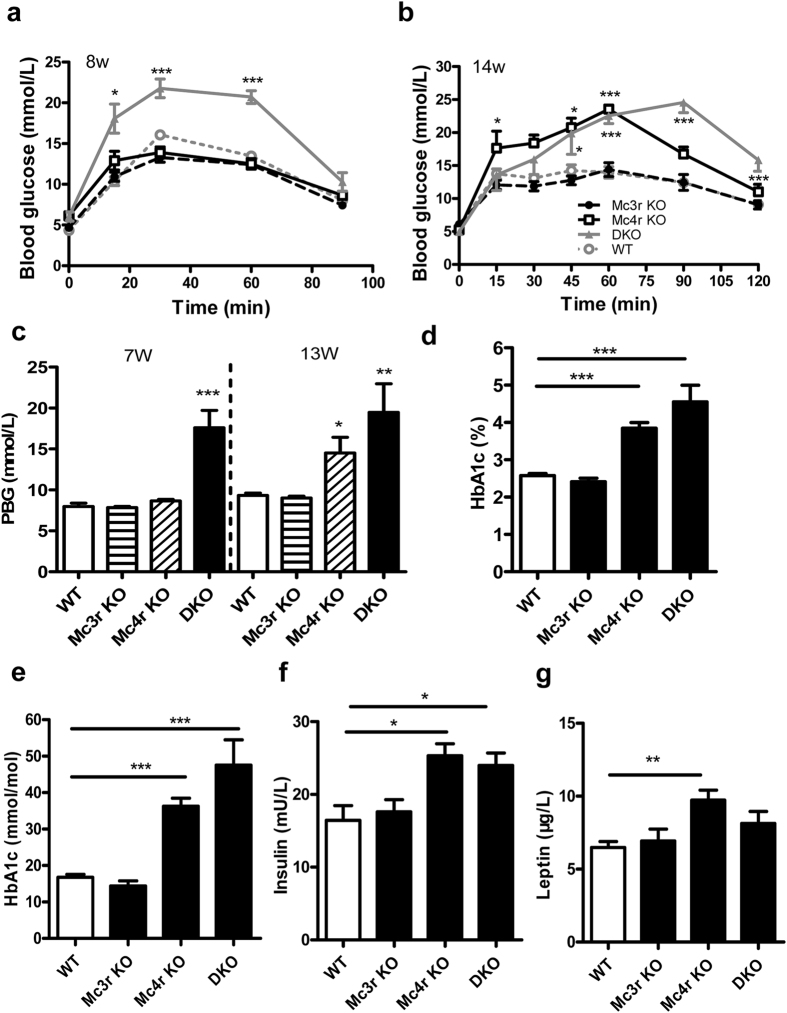
Effect of MC3R and MC4R deficiency on glucose metabolism. OGTT of different groups of rats at (**a**) 8 weeks and (**b**) 14 weeks of age. (**c**) Postprandial blood glucose (PBG) of mice at indicated ages. (**d**,**e**) Hb1Ac concentration, (**f**) Insulin and (**g**) Leptin levels of *Mc3r* KO, *Mc4r* KO, DKO and WT littermates at 14 week of age. Only data (mean ± SEM, n = 6–8) from males are shown. Females showed a similar trend. *p < 0.05, ***p < 0.001 vs. WT littermates (one-way ANOVA test).

When blood glucose homeostasis was evaluated in different groups of rats, we found that glucose tolerance states were unchanged in all *Mc3r* KO rats. At 8 weeks of age only DKO rats showed a reduced glucose tolerance, while both *Mc4r* KO and DKO rats demonstrated a reduced tolerance by the end of 14 weeks (Fig. 6a,b). We then evaluated whether there was any change in the postprandial blood glucose (PBG) level. Only DKO rats showed elevated PBG levels at 7 weeks (n = 8, p = 0.0008), but both *Mc4r* KO and DKO displayed hyperglycaemia by 13 weeks of age (Fig. 6c, n = 6, p = 0.02 for *Mc4r* KO, p = 0.002 for DKO). These results were partly in line with the previous report in which serum glucose was unchanged at 4–8 week old, but started to show hyperglycemia at 10–14 weeks of age in male *Mc4r* KO mice^8^, although in our study, both male and female *Mc4r* KO or DKO rats presented the same glucose metabolism characteristics. In addition, a significant elevation of glycosylated hemoglobin (HbA1C) was observed in both *Mc4r* KO and DKO rats at 14 weeks of age, whereas no change was manifested in *Mc3r* KO (Fig. 6d,e, n = 6–8, p = 0.0005 for *Mc4r* KO and p < 0.0001 for DKO). Similarly, insulin was elevated in both *Mc4r* KO and DKO rats at 14 weeks of age (Fig. 6f, n = 6–8, p = 0.01 for *Mc4r* KO, p = 0.04 for DKO). Leptin levels were also elevated in *Mc4r* KO as well as DKO rats, although the latter did not reach the significant level (Fig. 6g, n = 6–8, p = 0.007 in *Mc4r* KO, p = 0.07 for DKO). At the same time, *Mc3r* KO did not display any significant alteration in insulin or leptin levels (Fig. 6f,g). The above results suggested that although *Mc3r* KO did not show a significant change in glucose metabolism, it showed an additive effect on top of *Mc4r* KO and led to an earlier and more severe hyperglycaemia in DKO.

## Discussion

In this study, we report rat models of *Mc3r* total KO, *Mc4r* total KO and *Mc3r/Mc4r* DKO in a homogenous background, and provide a systematic comparison of MC3R and MC4R functions in energy metabolism. Our data reinforce previous reports about MC4R’s role in energy metabolism. However, for the first time MC3R deficiency was found to display a reduced body weight, whereas at the same time exhibited additive effects on top of MC4R deficiency in lipid and glucose metabolism. This report will be useful not only in understanding the effects of MC3R and MC4R deficiency in obesity and obesity-related diseases, but also provides better options of rat models that can be used in obesity and type 2 diabetes studies. Compared with other obesity rat models, our *Mc4r* KO and DKO manifested a relatively earlier hyperglycaemia on a normal chow diet (Table 1). Zucker rats have normal blood glucose^28^, while *Mc4r*^K314x^ rats were reported have to be maintained on a moderately high-fat diet, with a limited commercial availability^29^.

The effect of MC4R deficiency on obesity and metabolism homeostasis is fairly consistent. Compared with *Mc4r*^K314x^ rats, our *Mc4r* KO founders had a similar phenotype in body weight, food intake, insulin and leptin level^26^, which was further confirmed in this study. We speculate that our *Mc4r* KO rats may have a greater food intake increase if we use a chow diet with a higher fat content, as *Mc4r* KO mice were reported to show a fat-induced hyperphagia^30^,^31^. Additionally, our *Mc4r* KO rats have similar body size and hyperglycaemia phenotypic characteristics as *Mc4r* KO mice (Table 2). However, the function of MC3R is more complicated. Initial studies of the phenotypes of *Mc3r* KO mice indicated a very small body weight increase under chow diet, despite the increased fat mass^16^,^32^. In addition, there was no hyperphagia or glycaemia, indicating that *Mc3r* KO mice are more protected from metabolic syndrome compared to other obesity models with similar levels of adiposity^33^. More recent studies have demonstrated an exaggerated diet-induced obese phenotype in *Mc3r* KO^12^,^34^. However, while *Mc3r* KO fed on a high fat diet achieved a level of adiposity comparable to that observed in *Mc4r* KO mice, the insulin resistant phenotype remained modest and less severe^35^. While most of our data related to *Mc3r* KO were consistent with previous mouse data including increased adiposity, it is worth noting that there were some significant difference (Table 2). First, our *Mc3r* KO rats displayed a significant reduction in body weight and food intake as compared to WT littermates. Furthermore, from PND 45 to 85 (which is the usual age for rats used in biological research), DKO rats displayed almost the same body weight and weight gain as *Mc4r* KO rats. Previous reports stated that *Mc3r*/*Mc4r* DKO mice were significantly heavier than *Mc4r* single KO mice at the age of 26 weeks, although no data demonstrated whether any difference existed before this age^16^,^36^. Although it seems different from results in mice, the reduction or lack of additive effect in body weight of *Mc3r* deficient rats is consistent with the reported notion that MC3R is an inhibitory autoreceptor on proopiomelanocortin (POMC) neurons. This inhibitory role of MC3R was suggested when the MC3R agonist, D-trp^8^-γ-MSH (melanocyte stimulating hormone) was found to cause an increase in food intake which was absent in MC3R KO mice^37^. However, it is also possible that the reduced body size of our *Mc3r* KO rats is due to other reasons, such as reduced hunger sensations owing to an attenuated response of AgRP/Npy neurons^38^. There were no MC3R null humans. Although MC3R mutations had been reported to associate with obesity in human^17^,^18^,^19^,^39^,^40^, whether MC3R variants play a causative role still needs more investigation. Therefore it is urgent that the physiological function and signalling of MC3R be clarified. Up till now, the signal pathway of MC3R is not well defined. Results generated from various models were quite different. Some groups reported that MC3R coupled to adenylyl cyclases through Gs, leading to stimulation of cAMP production^12^,^41^. Others demonstrated calcium as well as MAP kinase activation in *ex vivo* or *in vivo* models^41^,^42^,^43^,^44^.

Despite the fact that MC3R and MC4R deficiency showed an opposite effect on food consumption and body weight in our mutant rats, we observed additive effects of *Mc3r* KO to *Mc4r* KO in phenotypes including adipocyte size, hepatic steatosis, lipid profile, OGTT and hyperglycaemia. This suggests that MC3R has a more complicated role in energy regulation. It may exert different functions on feeding behavior and lipid regulation through different signalling pathways. In fact, MC4R was recently found to exert distinct physiological functions through different G proteins. The research found that in addition to the effect of MC4R on glucose metabolism and energy expenditure which are mediated by Gαs, a pathway for appetite regulation was identified which was mediated by Gαq/11^45^. It is known that melanocortin receptors share some common natural agonists, but with different affinities and different signalling pathways. This maybe one of the reasons why MC3R and MC4R have additive effects in energy metabolism. In addition, both MC3R and MC4R are expressed in the CNS as well as in distinct peripheral sites such as gut, muscle, pancreas^13^,^46^,^47^. They had been reported to exert central and peripheral actions in energy metabolism^48^. It is very likely that MC3R and MC4R regulate adiposity and lipid metabolism using different mechanisms, and both of them together could more accurately regulate energy balance.

Our data confirmed the notion that MC3R has a critical role in inhibition of energy storage, and its ablation led to the increased body fat^16^,^32^. However, despite the obvious increase in body fat, we did not observe alterations in lipid contents of *Mc3r* KO rats, indicating that MC3R by itself does not have a dominant function in lipid metabolism. While *Mc3r* KO showed a subtle phenotype, DKO exhibited worse phenotypic features than single KO rats, suggesting that both receptors are important and non-redundant in energy balance and that some interactions probably exist between them. The phenotype of *Mc3r* KO might even be dependent upon the presence of MC4R. A number of MC3R and MC4R agonists have been developed, yet none of them has demonstrated satisfactory selectivity^12^,^49^. It is important to identify more selective agonists for MC3R and MC4R in order to elucidate their precise physiological functions *in vivo*. For obesity or diabetic drug development, it may be more attractive to look for pathway selective compounds of these receptors to regulate specific functions.

In conclusion, this study is the first systematic comparison of MC3R and MC4R single deficiency and double deficiency in rats with the same genetic background. It seems these two receptors possess non-redundant but somewhat overlapping functions in energy metabolism. Although MC3R deficiency led to a reduction of food consumption and body weight, it did show some additive effects on top of MC4R deficiency in both lipid and glucose metabolic disorders, which suggests different signalling pathways exist for MC3R. Data presented in this paper shed some new lights on the mechanism of MC3R and MC4R function in metabolic regulation and revealed potential interactions between MC3R and MC4R deficiency. The *Mc4r* KO and DKO rats generated in our lab will be beneficial for future studies to further elucidate MC3R and MC4R’s function and signalling pathways, and provide better rat models for novel anti-obesity or anti-diabetic drug development. In addition, the *Mc3r* KO rat might have its own value as a unique model in which high adiposity is not linked with body weight increase and insulin resistance.

## Methods

### Animals

Heritable total *Mc3r* or *Mc4r* gene knockout were generated using CRISPR-Cas9 system in our lab^26^, using Sprague Dawley (SD) rats from SLAC Laboratory Animal Co., Ltd. (Shanghai, China). The single KO homozygotes were breed from heterozygotes, and *Mc3r/Mc4r* DKO were generated by intercrossing double-heterozygous rats. The mutant rats appeared phenotypically normal except for changes in body weight and size. No obvious developmental and reproductive defects were noticed which was consistent with that reported in mouse models^50^. Animals were housed 2 per cage, maintained in a specific pathogen-free facility on 12-hour light/12-hour dark cycles at a constant room temperature (22 ± 1 °C), with free access to water and chow diet (18% protein and 6% fat, Xietong Medical and Biological Engineering Co. Ltd., Jiangsu, China). Food and water were weighed before and after each change, and the difference was calculated as estimated intake. Minimal bedding was used for easy inspection of possible abnormal spillage, which was not observed. In fact spillage was very little and similar in all four genotypes. All animal experimental procedures and techniques were approved by the Animal Ethics Committee of East China Normal University (Permit number: R20151504), and methods were carried out in accordance with the approved guidelines.

### Genotype of *Mc3r* and *Mc4r* KO rats

Tail clips were subjected to a standard DNA extraction procedure. Identification of the *Mc4r* mutation was done by PCR, with primers 5′-GTCCGCCACAGCCAGACTAC-3′ (sense) and 5′-CGCTGCTTCTGACCCTGTTC-3′ (antisense). The *Mc3r* mutation was identified by PCR with primers 5′-CCCAGCAGCTTGCTCAGGAC-3′ (sense) and 5′-CTCCAGGGAGTTGGACAGGC-3′ (antisense), together with bpm1 (New England Biolabs Inc., USA) digestion analysis. Mutations in *Mc3r* and *Mc4r* were successfully transmitted to the following generations. The genotypes of all of the rats were verified as correct.

### Quantitative real-time PCR analysis

Rats were sacrificed and total RNA was isolated from brain, liver and WAT with TRIzol (Invitrogen). Possible DNA contamination was eliminated using DNase I (Amplification Grade, Invitrogen). The purified RNA was then reverse-transcribed to cDNA using the Prime Script RT kit (Takara). Real-time PCR was performed in quadruplicate with a SYBR Green PCR Master Mix (Takara) according to the manufacturer’s instruction and ran on the MX3005p system (Stratagene, USA). Data were calculated through MXProv4.1. (Stratagene, USA). Primers (synthesized by Shanghai Biosune, China) were as follows: *Mc3r*, (forward) 5′-TGCTGCCCGTCCTCCTCTTA-3′ and (reverse) 5′-CCAGGATCACCAGGATGTTTT-3′; *Mc4r*, (forward) 5′-GGACCACTTCAAGGAGGATT-3′ and (reverse) 5′-CACCCAGAGTCACAAACACC-3′; the reference gene GAPDH, (forward) 5′-TCTCTGCTCCTCCCTGTTCT-3′ and (reverse) 5′-TACGGCCAAATCCGTTCACA-3′.

### Bodyweight, size and Lee’s index calculation

Weight gain was monitored throughout the study. For animal size, rats were anesthetized with isoflurane (Hebei Yipin Pharmaceutical Co. Ltd, Hebei, China) and body length (from anus to nose) was measured. Because there is possible body length change in the mutant rats, we used Lee’s index as an additional measurement for the degree of obesity. Lee’s index was calculated as bodyweight (gram)^1/3^/body length (cm) × 1000^51^.

### Biochemical analysis

Blood was collected from the retro-orbital plexus and serum was obtained by centrifugation at 3000 rpm for 15 minutes at 4 ^o^C, which was kept frozen at −80 ^o^C until analysis. Serum triglyceride, total cholesterol, aspartate aminotransferase, alanine aminotransferase, uric acid, creatinine, LDL-c and HDL-c were analyzed using the AU680 Automatic Biochemistry Analyzer (Beckman Coulter, USA). Serum insulin, leptin and free fatty acid levels were measured using ELISA kits (R&D systems, USA). For HbA1c measurement, plasma samples were tested in a rat HbA1c kit (Shensuo Youfu Medical Diagnosis Products Co. Ltd, Shanghai, China) according to the manufacturer’s instruction. PBG levels of overnight fasted rats were measured 2 h after an oral glucose challenge at 2 g/kg, using a portable glucose meter (ACCU-CHEK Performa Nano, Roche).

### Oral Glucose Tolerance Test (OGTT)

Animals were fasted overnight and blood glucose was measured from tail bleeds before and 15, 30, 45, 60, 90, and 120 minutes after intragastric administration of 50% glucose (2.5 g/kg), using the Roche glucose meter.

### Hematoxylin-Eosin (HE) staining

Rats were sacrificed by CO2 inhalation. Visceral adipose tissue, liver and kidney samples were collected and fixed overnight in ice-cold 4% paraformaldehyde solution, then embedded in paraffin. Serial 5 μm sections were cut and stained with hematoxylin-eosin for histological analysis. Images were captured on a Leica DM4000 B LED microscope with a Leica DFC310FX Camera and software kit. Quantification of HE staining was done by Image-Pro^®^ Plus version 6.0 software. A minimum of 5 independent fields per sample was evaluated.

### Oil Red-O staining

Liver samples were fixed overnight in 4% paraformaldehyde, followed by immersion in 15% and then 30% sucrose. Serial 5 μm sections were cut and stained with the Oil Red-O staining kit (Jiancheng Scientific Inc., Nanjing, China) for 3~5 minutes in accordance with the manufacturer’s instruction, and finally counterstained with hematoxylin. Relative areas of lipid accumulation (expressed as percentage Oil Red-O staining) were quantified using Image-Pro^®^ Plus version 6.0 software. A minimum of 5 independent fields per sample was evaluated.

### Statistical analyses

Data are expressed as mean ± SEM. One way ANOVA followed by Turkey’s multiple comparisons test was applied to analyse differences among groups, except for body weight (Fig. 1a,b and Supplementary Fig. S2), where the comparisons were analysed using repeated measures analysis followed by Bonferroni post-tests. p < 0.05 was considered to be statistically significant.

## Additional Information

**How to cite this article**: You, P. *et al*. Effects of Melanocortin 3 and 4 Receptor Deficiency on Energy Homeostasis in Rats. *Sci. Rep*. **6**, 34938; doi: 10.1038/srep34938 (2016).

## Supplementary Material

Supplementary Information

## Figures and Tables

**Table 1:** Comparison of our *Mc4r* KO and DKO rats with other obesity rat models.

	*Mc4r* KO	DKO	*Mc4r*^K314x^	Zucker	*Lepr*^−/−^52
Obesity	appears at 4–6 weeks	appears at 4–6 weeks	appears at 4 weeks^25^	appears at 4 weeks	appears at 4 weeks
Hyperphagia	yes	yes	yes	yes	yes
Blood glucose	hyperglycaemia at 12 weeks	hyperglycaemia at 8 weeks	—	normal blood glucose^28^	hyperglycaemia only observed in males
Glucose intolerance	appears at 14 weeks	appears at 8 weeks	—	delayed onset^53^	appears at 8 weeks
Lipid profile	high lipid content	higher than *Mc4r* KO	—	—	—
Diabetic complication	liver steatosis	liver steatosis	—	renal^54^	pancreas, liver and renal lesion

**Table 2:** Comparison of previous *Mcr* KO mice and our *Mcr* KO rats.

	*Mc3r* KO rats	*Mc4r* KO rats	DKO rats	*Mc3r* KO mice^16^	*Mc4r* KO mice^8^,^50^	DKO mice
Body weight	reduced	increased	Increased, same level as *Mc4r* KO	slight increase after 24 weeks	increased	increased, more than *Mc4r* KO at 26 weeks^16^
Fat mass	increased	increased	increased	increased	increased	—
Body length	reduced	increased	increased	reduced	increased	—
Hyperphagia	hypophagia	yes	yes	hypophagia	yes	—
Glucose	normal	T2D	T2D	normal	T2D	—
Insulin	normal	increased	increased	mild increase at 24 weeks	increased	—
Leptin	normal	increased	mild increase	mild increase at 24 weeks	increased	—
