# Supplementary material for: Effects of Melanocortin 3 and 4 Receptor Deficiency on Energy Homeostasis in Rats
Source: Sci Rep. 2016 Oct 7;6:34938. doi: 10.1038/srep34938 (PMC5054679; doi:10.1038/srep34938)

Effects of Melanocortin 3 and 4 Receptor Deficiency on Energy Homeostasis in Rats

Panpan You, Handan Hu, Yuting Chen, Yongliang Zhao, Yiqing Yang, Tongtong Wang, Roumei Xing, Yanjiao Shao, Wen Zhang, Dali Li, Huaqing Chen, Mingyao Liu

Supplementary information

Supplementary Figure S1. Identification of the *Mc4r* and *Mc3r* mutations. (**a**) Genotype of *Mc3r* and *Mc4r* KO rats. (**b**) DNA sequences of *Mc3r* and *Mc4r* genomic loci in mutants. The change in the base-pair sequence is shown at right of the sequences. (**c**) mRNA levels of *Mc3r* and *Mc4r* in liver, brain and WAT of WT and KO rats.

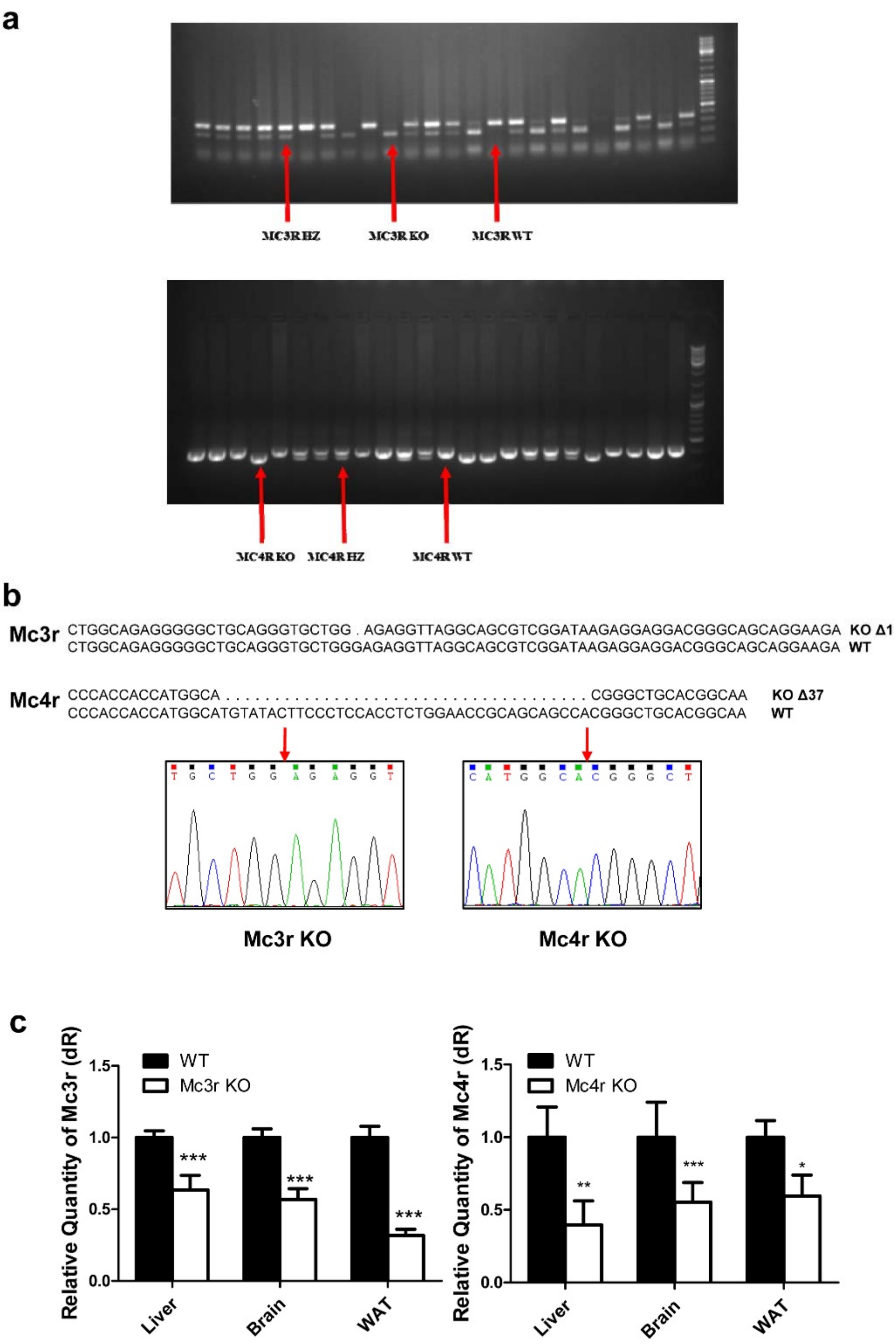

Supplementary Figure S2. Effect of MC4R or MC3R single deficiency on body weight. (**a, b**) Body weight and (**c, d**) Relative body weight were measured from 4 weeks to 18 weeks for *Mc3r* KO, *Mc4r* KO and WT littermates (n = 8). Data are shown as mean ± SEM. ***$p < 0.001$ vs. WT control (repeated measures analysis test followed by Bonferroni post-tests).

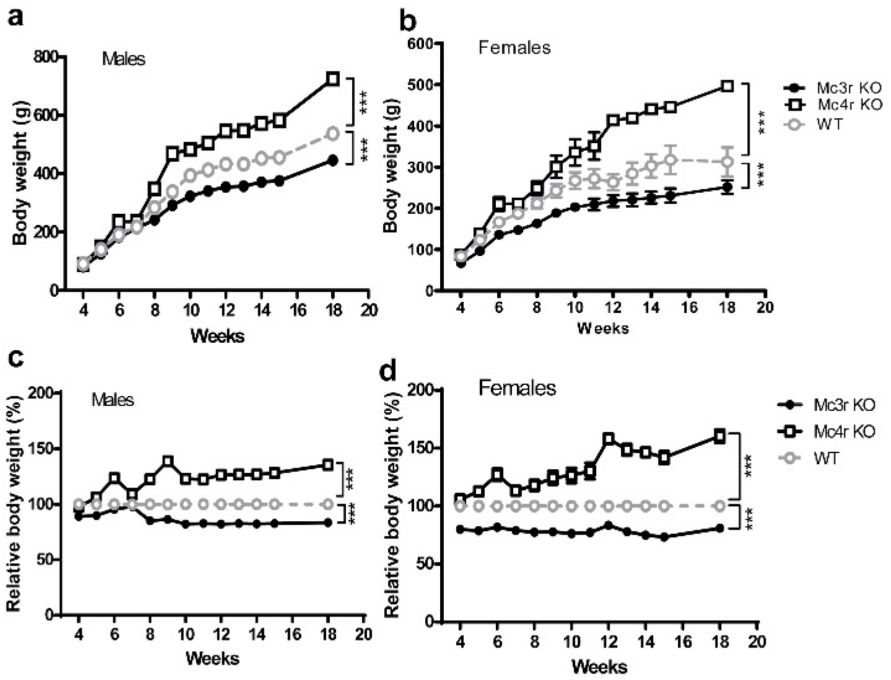

Supplementary Figure S3. Effect of MC3R and MC4R deficiency on kidney. Relative kidney size (vs. body length) (**a**), Uric acid (**b**) and Creatinine (**c**) were measured at 14 weeks of age. Data are shown as mean ± SEM (n = 4-8). **p < 0.01, ***p < 0.001 vs. WT control (one-way ANOVA test). (**d**) Representative image of kidney from at least 3 animals of different groups (magnification ×100 or × 400).

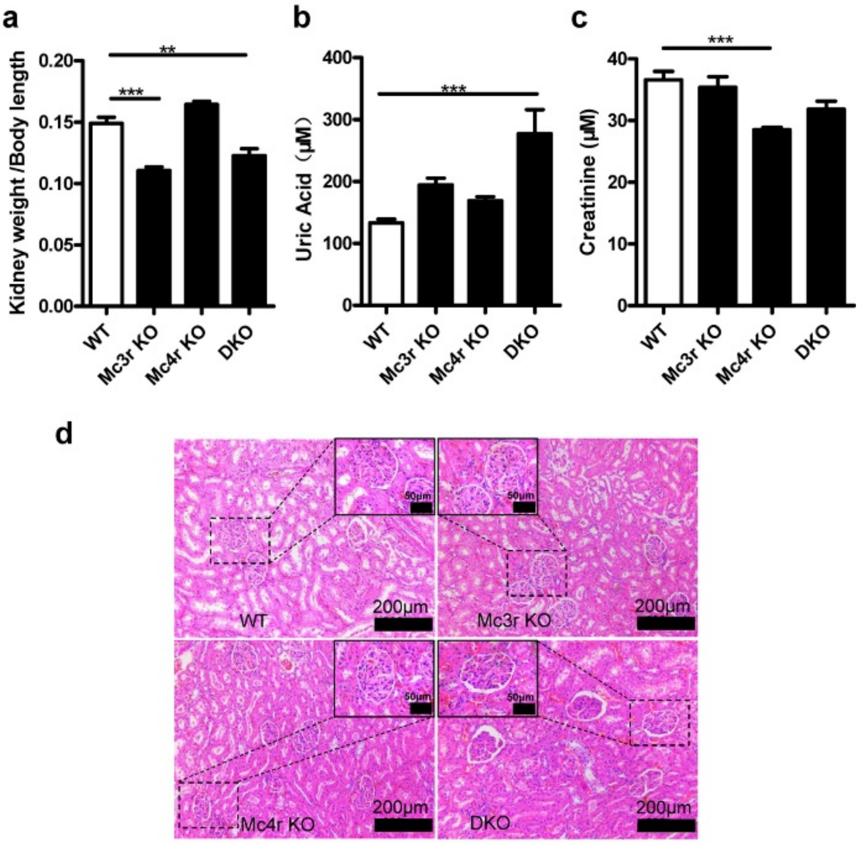

Supplement: Supplementary Information [file srep34938-s1.pdf]
